# Impact of prebiopsy MRI on prostate cancer staging: Results from the Norwegian Prostate Cancer Registry

**DOI:** 10.1002/bco2.214

**Published:** 2023-01-10

**Authors:** Erik Skaaheim Haug, Tor Åge Myklebust, Patrick Juliebø‐Jones, Lars Anders Rokne Reisæter, Kirsti Aas, Arne Stenrud Berg, Christoph Müller, Bjørn Hofmann, Øystein Størkersen, Kim L. Nilsen, Tom Børge Johannesen, Christian Beisland

**Affiliations:** ^1^ Department of Urology Vestfold Hospital Trust Tønsberg Norway; ^2^ Institute of Cancer Genomics and Informatics Oslo University Hospital Oslo Norway; ^3^ Cancer Registry of Norway Oslo Norway; ^4^ Department of Urology Haukeland University Hospital Bergen Norway; ^5^ Department of Clinical Medicine (K1) University of Bergen Bergen Norway; ^6^ Helse Bergen HF, Department of Radiology Haukeland University Hospital Bergen Norway; ^7^ Department of Urology Oslo University Hospital Oslo Norway; ^8^ Department of Oncology Drammen Hospital Drammen Norway; ^9^ Department of Oncology, Cancer Treatment Centre Sørlandet Hospital Kristiansand Norway; ^10^ Department of Health Sciences Norwegian University of Science and Technology Gjøvik Norway; ^11^ Centre for Medical Ethics University of Oslo Oslo Norway; ^12^ Department of Pathology, St. Olavs Hospital Trondheim University Hospital Trondheim Norway

**Keywords:** diagnosis, MRI, outcome, population based, prostate cancer, risk groups, staging, TNM, treatment

## Abstract

**Objectives:**

The aim of this study is to evaluate the 2015 introduction of prebiopsy magnetic resonance imaging of the prostate (MRI‐P) as the standard of care for diagnosing prostate cancer (PCa) by the Norwegian public health care authorities. There were three specific objectives of this study: first, to evaluate the consequences of using different TNM manuals for clinical T‐staging (cT‐staging) in a national setting; second, to determine if the data reveals that MRI‐P based cT‐staging is superior to digital rectal examination (DRE)‐based cT‐staging compared with pathological T‐stage (pT‐stage) post radical prostatectomy; and third, to assess whether treatment allocations have changed over time.

**Materials and Methods:**

All patients registered in the Norwegian Prostate Cancer Registry between 2004 and 2021 were retrieved and 5538 were eligible for inclusion. Concordance between clinical T‐stage (cT‐stage) and pT‐stage was assessed by percentage agreement, Cohen's kappa and Gwet's agreement.

**Results:**

MR visualisation of lesions influences reporting of tumour extension beyond DRE findings. Agreement between cT‐stage and pT‐stage declined from 2004 to 2009, which coincided with an increase in the percentage being pT3. From 2010, agreement increased, which aligned with changes in cT‐staging and the introduction of MRI‐P. From 2017, regarding the reporting of cT‐DRE and cT‐Total (overall cT‐stage), agreement diminished for cT‐DRE but remained relatively stable (>60%) for cT‐Total. Regarding treatment allocation, the study suggests that staging with MRI‐P has shifted treatment towards radiotherapy in locally advanced high‐risk disease.

**Conclusion:**

Introduction of MRI‐P has affected cT‐stage reporting. Agreement between cT‐stage and pT‐stage appears to have improved. This study suggests that use of MRI‐P influences treatment decisions in certain patient subgroups.

## 1 INTRODUCTION

Risk stratification of prostate cancer (PCa) has been traditionally based on biopsy grade, PSA and clinical T‐stage (cT‐stage).^1^ However, there is a well‐documented discordance between cT‐stage and pathological T‐stage (pT‐stage), which is often attributed to the lower accuracy of digital rectal examination (DRE) for extra‐prostatic extension (EPE).^2^ In contrast, magnetic resonance imaging of the prostate (MRI‐P) has a higher sensitivity for EPE^2^ and should therefore improve staging accuracy. Recent studies have highlighted the potential benefits of MRI‐P such as avoiding unnecessary biopsies.^3^, ^4^ To this end, Norwegian public health care introduced prebiopsy MRI‐P as the national standard in 2015.

As a result of this change to the diagnostic pathway, radiographic depiction of poor prognostic factors such as EPE became available to clinicians. This may have influenced clinicians' pretreatment interpretation of risk and clinical staging. Potential sequelae included an increased risk of upstaging and subsequent overtreatment.^2^, ^3^ Six years later, it remains unclear to what extent prebiopsy MRI‐P has affected clinical staging and treatment choices at a population level.

To investigate this further, we carried out a study under the auspices of the Norwegian Prostate Cancer Registry (NPCR), which is integrated into the Cancer Registry of Norway (CRN). Three main aims were established: first, to evaluate any consequences on a national level of using different TNM manual editions for cT‐staging; second, to determine if the data supports the premise that MRI‐P based cT‐staging is superior to DRE compared with pT‐stage after radical prostatectomy (RP); and finally, to identify changes in treatment allocation after mandatory prebiopsy MRI‐P was introduced.

## 2 MATERIALS AND METHODS

### 2.1 PCa care in Norway

Over 95% of new PCa diagnoses in Norway are treated within the public health care system. Between 2004 and 2016, a 36% increase in the absolute number of PCa diagnoses was recorded (*n* = 3849 and *n* = 5233, respectively).^5^ This upsurge was likely due to opportunistic PSA testing and earlier disease detection. Meanwhile there was a disproportionate 630% increase in the annual number of recorded RPs (*n* = 272 and *n* = 1720, respectively).^5^ However, this change was likely the result of a revised treatment policy whereby radical surgery was extended to higher risk disease. Furthermore, RP was increasingly offered to men >70 years.

In 2015, the Norwegian Ministry of Health introduced national cancer pathways to improve quality standards. Prebiopsy MRI‐P became mandatory and delivery of care by designated regional centres, which met defined standards of expertise and case volume (>50 RPs annually).

### 2.2 Patient registration and inclusion

Norwegian law requires all new cancer diagnoses to be reported to the CRN. This database has a 98% coverage for diagnostic data and surgery.^5^, ^6^ This study required neither patient consent nor ethical approval as it was under the remit of CRN/NPCR.

All PCa patients registered in CRN from 2004 to 2021 were retrieved from the registry (*n* = 82 015). Exclusion criteria included incidental diagnosis at TURP (*n* = 4788), missing cT‐DRE (*n* = 7335), RP not within 180 days of diagnosis (*n* = 50 506), missing pT (*n* = 1265) information, diagnosis before 1 July 2017 (*n* = 12 248) and missing cT‐MRI or cT‐Total (*n* = 299). Five thousand five hundred thirty‐eight patients were eligible for inclusion in the final analysis (Figure S1).

### 2.3 Methodology used for the study

### 2.4 cT‐staging and pT‐staging

cT‐stage at the time of diagnosis has been reported to NPCR since it was established in 2004. Prior to 2010, the sixth TNM manual was used for staging, which only included DRE.^7^ Between 2010 and 2017, cT‐stages were reported according to the seventh TNM manual, and therefore, cT‐stage could be based on DRE and imaging. This edition outlined that stage cT2 should be either ‘palpable’ or ‘reliably visible’ but no further specifications were made.^8^ If neither were present, the tumour would be staged cT1c, even with bilateral positive biopsies. However, owing to uncertainties surrounding interobserver reproducibility, patient selection and contradictory results, the eighth TNM classification (2017) states that cT‐stage should no longer be based on imaging.^9^, ^10^ To this effect, the reporting of cT‐stage to NPCR has been based on DRE alone (cT‐DRE) since 1 June 2017. However, it should be noted that in this study, pT‐stage has been reported according to the corresponding TNM edition. Only T2 and T3 without subcategories have been used.

### 2.5 MRI‐P data

MRI‐P was introduced to clinical practice in Norway in 2007 and was already in widespread use by the time of the 2015 cancer pathway recommendations. Figure S2 displays the increasing trend of MRI‐P utilisation. Since 2015, over 12 000 MRI examinations were performed across the public and private sectors annually in Norway. Separate T‐staging according to MRI‐P was not reported to NPCR until 1 July 2017. Thereafter, separate cT‐staging based on MRI‐P became part of the registry for lesions classified as PI‐RADS > 3.^11^ This is an adaption to the previous concept of ‘reliably visible’ in the seventh TNM manual. Consequently, PI‐RADS ≤ 3 lesions are considered stage cT1.

Based on all available data (imaging, laboratory tests, histology and clinical examination), an overall clinical cT‐stage (cT‐Total) is also reported to register the clinician's overall perception of the TNM‐staging system. Staging source is also recorded (e.g., is it a multidisciplinary team [MDT] or a single physician decision).

### 2.6 Grading and risk groups

NPCR uses the International Society of Urological Pathology (ISUP) 2014 grade group (GG) system for tumour aggressiveness.^12^, ^13^ Patients are allocated to risk groups according to the European Association of Urology (EAU) risk groups for biochemical recurrence of localised and locally advanced PCa.^14^ EAU guidelines specifically state that risk stratification should be based on cT‐stage by DRE.^15^ However, risk groups in this study have also been calculated based on cT‐DRE and cT‐Total to determine the impact of MRI‐P.

### 2.7 Data retrieval and statistics

Descriptive statistics were presented using absolute and relative frequencies. When deemed suitable, we calculated the corresponding 95% confidence interval (CI). When assessing agreement between cT‐stage and pT‐stage, we calculated the percentage agreement, Cohen's kappa and Gwet's agreement coefficient (AC1) and their respective 95% CI. The latter was included to complement the kappa coefficient, which might be influenced by low/high prevalence in one or more of the cT‐stage and/or pT‐stage.^16^ All analyses were done using Stata version 17.0 and performed by professional statistician (TAM) employed by CRN. All agreement statistics were calculated using the user‐written command kappa.^17^

## 3 RESULTS

### 3.1 Reporting of cT‐stage in the NPCR

**FIGURE 1:**
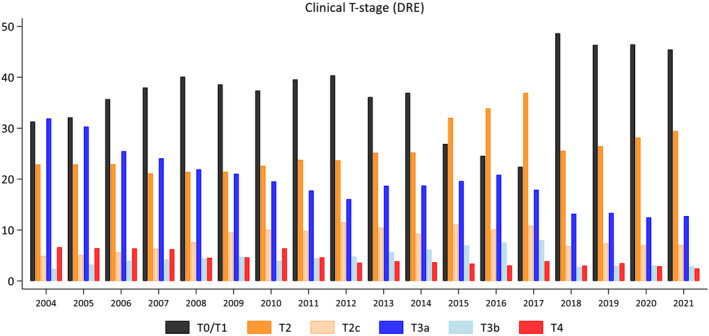
Distribution of reported DRE‐based clinical T‐stage (cT‐stage) in Norwegian men diagnosed with prostate cancer during the period 2004 to 2021

Figure 1 demonstrates the development in DRE‐based cT‐stage reporting during the study period. In the early period (2004–2009), as the incidence of PCa increased, the percentage of cT3 and cT2 dropped with a corresponding increase in reported cT1. Between 2010 and 2014, there was an overall increase in reported cT2 stages. From 2015 to 2017, reported cT2 increased markedly, while reported cT1 diminished. From 2018 to 2021, reported cT1 increases, while cT2 and especially cT3 decreased.

### 3.2 Agreement between reported cT‐stage and pT‐stage over time

**FIGURE 2:**
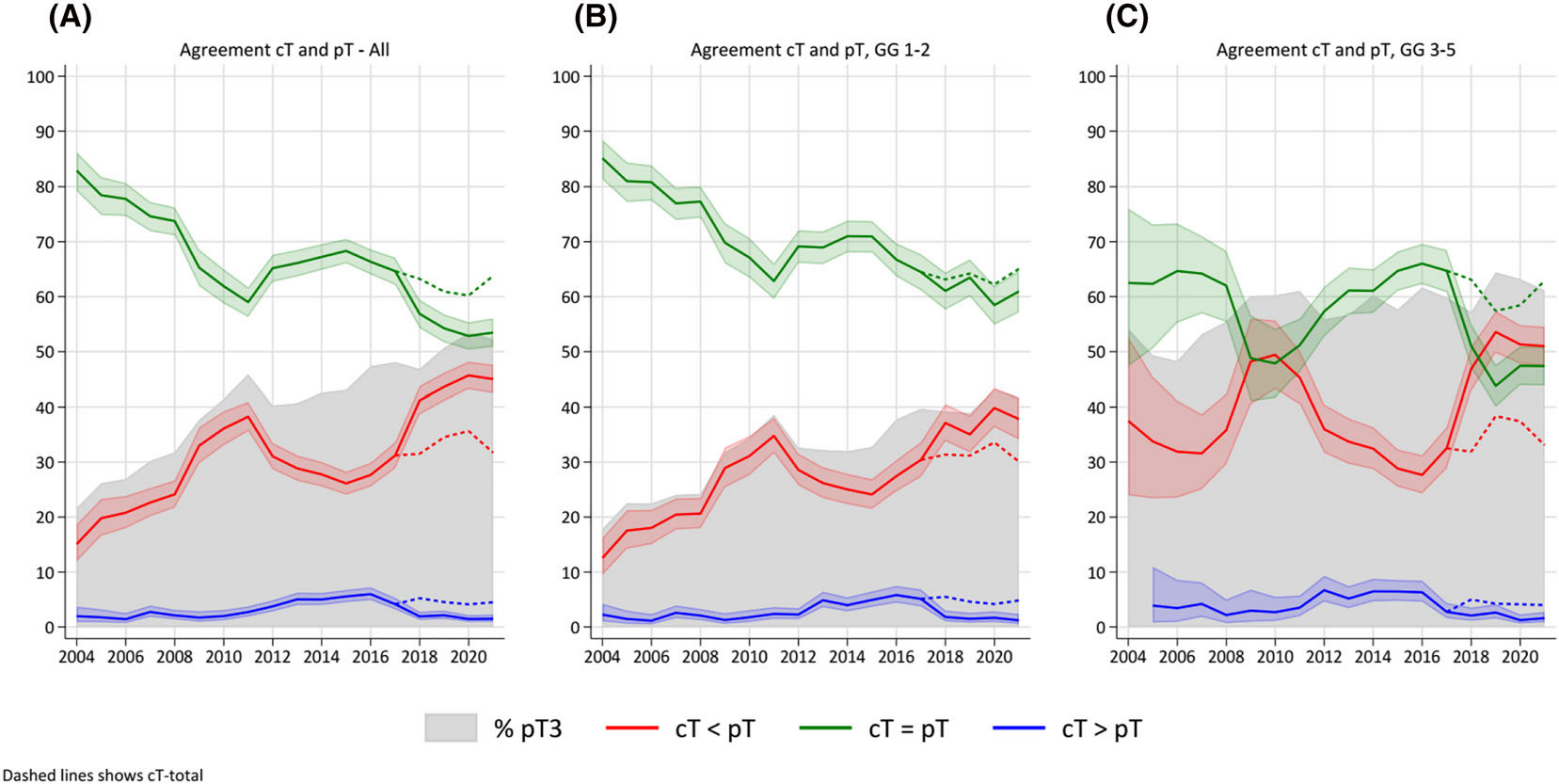
(A) Agreement of reported clinical T‐stage (cT‐stage) and pT after radical prostatectomy in Norway from 2004 (*n* = 272) to 2021 (*n* = 1792). From 2017, the reported cT‐stage is based on DRE only (cT‐DRE) (continuous lines). Dashed lines from 2017 represents cT‐stage based on DRE and MRI (cT‐Total). (B) Same as (A) but for ISUP grade groups (GGs) 1 and 2. (C) Same as (A) but for ISUP GG ≥3

Figure 2A shows the agreement between reported cT‐stage and pT‐stage over the study period. The agreement declined during the early years, which coincided with an increase in the percentage being pT3. From 2010, degree of agreement increased, which aligns with both change in cT‐staging and introduction of MRI‐P. From 2017, regarding cT‐DRE and cT‐Total reporting, agreement diminished for cT‐DRE but remained at a relatively stable level (>60%) for cT‐Total.

The declining agreement of cT‐stage with pT in the early phase was most obvious for GGs 1 and 2, indicating a low predictive value for pT2 of a negative DRE (Figure 2B).

In GG ≥3, agreement between cT‐stage and pT‐stage improved with the introduction of MRI‐P. However, agreement of cT‐DRE and pT fell to less than 50% after 2017 when cT‐DRE and cT‐MRI and cT‐Total were reported separately. However, agreement remained >60% among the operated patients when using cT‐Total, as illustrated by the dashed lines in Figure 2A–C. Overall, MRI‐P seems to have affected agreement for GG ≥3 more than GGs 1 and 2.

When MRI‐P was introduced, overstaging (cT > pT) increased to approximately 5% for both GG ≥3 and GGs 1 and 2. However, it appears that overstaging has decreased for GG ≥3 after 2017 but remains stable for GGs 1 and 2.

### 3.3 Agreement of cT‐DRE, cT‐MRI and cT‐Total versus pT‐stage after inclusion of MRI‐P data in NPCR

In the final period (2017–2021), 49% of the patients had a palpable tumour on DRE, while 80% had a visible tumour (defined as PI‐RADS > 3) on MRI. There was superior agreement between cT‐Total and cT‐MRI compared with cT‐DRE. For cT‐DRE and cT‐MRI versus cT‐Total, Cohen's kappa was 0.48 (95% CI: 0.46–0.51) and 0.84 (95% CI: 0.82–0.86), respectively (Table 1a). Between the different cT‐staging methods and pT‐stage, overall reliability was poorer. Agreement between DRE cT and pT (54.4%, 95% CI: 53.1–55.7) was lower than for MRI‐based clinical staging (62.4%, 95% CI: 61.1–63.7) with agreement for cT‐Total almost identical (62.7%, 95% CI: 61.4–64.0; Cohen's kappa 0.27, 95% CI: 0.25–0.29) (Table 1b).

**TABLE 1a:** Agreement between cT‐Total and cT based on MRI and DRE

	# cT2‐Total (%)	# cT3‐Total (%)	Per cent agreement (95% CI)	Gwet's AC1 (95% CI)	Kappa (95% CI)
cT2 (DRE)	4132 (83.8)	802 (16.2)			
cT3 (DRE)	55 (9.1)	549 (90.9)	84.5 (83.6–85.5)	0.78 (0.77–0.80)	0.48 (0.46–0.51)
cT2 (MR)	3966 (56.9)	115 (43.1)			
cT3 (MR)	221 (15.2)	1236 (84.8)	93.9 (93.3–94.6)	0.90 (0.89–0.91)	0.84 (0.82–0.86)

**TABLE 1b:** Cohen's kappa and per cent agreement with pT after radical prostatectomy for cT based on DRE, MRI and both (Total)

	# pT2 (%)	# pT3 (%)	Per cent agreement (95% CI)	Gwet's AC1 (95% CI)	Kappa (95% CI)
cT2 (DRE)	2526 (51.2)	2408 (48.8)			
cT3 (DRE)	119 (19.7)	485 (80.3)	54.4 (53.1–55.7)	0.20 (0.17–0.23)	0.12 (0.10–0.13)
cT2 (MR)	2321 (56.9)	1760 (43.1)			
cT3 (MR)	324 (22.2)	1133 (77.8)	62.4 (61.1–63.7)	0.28 (0.25–0.31)	0.26 (0.24–0.28)
cT2 (Total)	2383 (56.9)	1804 (43.1)			
cT3 (Total)	262 (19.4)	1089 (80.6)	62.7 (61.4–64.0)	0.29 (0.27–0.32)	0.27 (0.25–0.29)

### 3.4 Changes in treatment allocation

**FIGURE 3:**
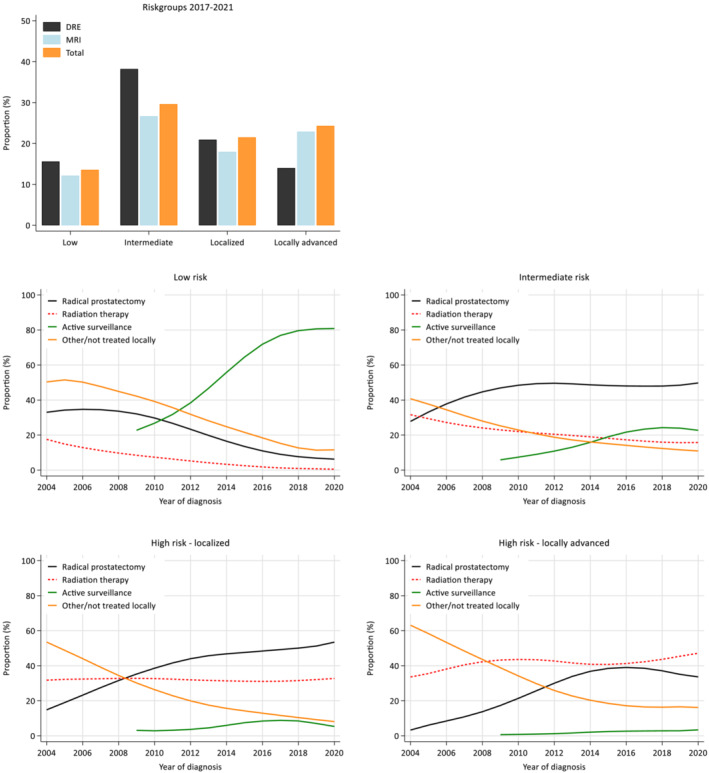
(A) European Association of Urology Risk Group (EAU RG) allocation for all patients diagnosed with Prostate Cancer in Norway between 2017 and 2021, based on different clinical T‐staging (DRE, MRI or Total). (B) Development in chosen primary treatment for the different EAU RGs during the period 2004–2021

Figure 3A demonstrates how the different methods of reporting cT‐stage affect risk group allocation. cT‐MRI and cT‐Total shift the patients towards the higher risk groups.

Figure 3B illustrates the evolution in allocated primary treatments over the study period. Several patterns can be seen, which are consistent with those reported elsewhere: first, the recommended transition from radical treatment towards active surveillance (AS) in the low‐risk group (currently >80%); secondly, the increasing implementation of AS in the intermediate‐risk group. In addition, a declining proportion of patients have not received local treatment, across all risk groups.

Regarding observed trends since the 2015 recommendations, for the locally advanced high‐risk category, the group most likely affected by MRI‐P, the proportion receiving radiotherapy has increased compared with surgery. In contrast, the percentage undergoing surgery continues to rise in the localised high‐risk group. Furthermore, the proportion being offered AS in the intermediate‐risk group seems to have plateaued.

## 4 DISCUSSION

### 4.1 Impact of MRI‐P on clinical staging

This study demonstrates that Norwegian clinicians refer to both the current TNM edition and NPCR specifications during mandatory reporting. Our findings show how the seventh TNM manual influenced cT reporting. Since MRI‐P was introduced, the proportion reported as cT1 has decreased. Reporting also significantly changed when MRI‐P became mandatory and staging was performed by the MDT.

Many clinicians feel that DRE holds limited predictive value regarding tumour extent. However, if DRE indicates an advanced clinical stage, the association with PCa ≥ pT3 or positive lymph nodes is present.^18^ However, Prebay et al. claim the predictive value of cT2 differed little from cT1c, which demonstrates the poor reliability of DRE.^18^ Clinicians tend to adopt the method deemed most reliable, even if the principle of TNM staging is to choose the lower stage if unclear. Currently, cT‐MRI is usually available to the clinician when performing DRE. The recent change in the eighth TNM edition,^15^ limiting cT‐staging to DRE, disrupts the TNM principle that all available tools should be used for staging pretreatment. The immediate change in reported cT‐DRE after the 2017 version (reporting of cT‐DRE and cT‐MRI separately) strongly implies clinician awareness of the difference between the modalities. The observation that cT by MRI‐P and cT‐Total showed greater similarity probably indicates that clinicians seem to have greater confidence in MRI‐P than DRE if there is uncertainty when determining overall stage (cT‐Total).

Reasons for not incorporating MRI‐P in staging are variations in availability and quality of MRI‐P as well as risk of stage migration.^19^ The latter is a substantial problem as the PCa population has changed as highlighted by the shift in staging between the SPCG4^20^ and PIVOT^21^ studies. Further changes are anticipated when recommendations based on studies such as PRECISION^4^ and PROMIS^22^ are translated into daily practice.

Current risk classifications do not consider MRI‐P findings. As demonstrated in Figure 3A and previous research,^3^ cT‐staging based on MRI will affect risk group distribution. Studies have demonstrated that including MRI‐P findings may improve prognostication^23^, ^24^ and there is an urgent need for updated risk classifications based on populations undergoing MRI‐P.^25^

### 4.2 Coherence with pT of cT by DRE and MRI‐P

Overall coherence of cT‐stage by DRE with pT‐stage after RP fell from 85% in 2004 to <60 % in 2011 (Figure 2A). This was most likely due to the increasing number of RPs performed on pT3 tumours. This trend is particularly evident for GGs 1 and 2 (Figure 2B), and the initial high correspondence was due to the low number of pT3 tumours. This demonstrates the low accuracy of DRE for cT1 and cT2. For GG ≥3, agreement was initially lower and decreased further as the proportion of pT3 approached 60%.

With increased use of MRI‐P, overall agreement climbed to almost 70% from 2012 to 2017. While the trend was evident for GGs 1 and 2 and GG ≥3, the impact was largest in the latter. Of note, during that period, overstaging increased markedly and almost reached 5%. This reinforces that MRI‐P is not a perfect tool for distinguishing T2 from T3.^26^, ^27^, ^28^

From 2017, with separate reporting of cT‐DRE, cT‐MRI and cT‐Total, the overall agreement of cT‐DRE and pT fell. However, when using cT‐Total including MRI, agreement remained relatively stable within high Gleason group. A likely interpretation is that the apparent improvement between 2010 and 2017 is the effect of clinicians reporting cT‐stage based on a combination of imaging (MRI‐P) and DRE, such was allowed for in the seventh TNM manual. The present study demonstrates that staging based on MRI‐P is improved and increases agreement between cT and pT after RP by approximately 20%. While this is consistent with previous research,^3^ this is the first study using data from a nonselected population at a national level.

### 4.3 Clinical significance of MRI‐P implementation

Draulans et al. showed that MRI‐P significantly changed treatment recommendations.^3^ Firm conclusions cannot be drawn from our registry‐based study. However, it interesting that the number of locally advanced high‐risk patients undergoing surgery declined after 2016. This suggests that staging with MRI‐P has shifted treatment towards radiotherapy in locally advanced high‐risk disease. A steady reduction of positive surgical margins for pT3 tumours during the same period,^5^ supports this conclusion and indicates both improved selection between radiotherapy and surgery, as well as better treatment planning. Furthermore, the 20% increase in AS among intermediate‐risk patients is likely accounted for by MRI‐P to a certain extent.

### 4.4 Strengths and limitations

The study aimed not to evaluate accuracy of MRI‐P for cT‐staging but rather to determine overall effect of an additional instrument. Data on MRI protocols in NPCR are limited. Practice patterns vary, and it is possible that some patients had additional imaging preoperatively. Over time, changes in EPE interpretation may have impacted the observed agreement between cT‐stage and pT‐stage. Extent of EPE has also not been evaluated in this study. Given that it is beyond the discriminative ability of both DRE and MRI to characterise minimal EPE, the agreement cannot be perfect.

However, a strength is that this study reflects clinical practice on a national level, outside of a trial setting. Although not all changes in clinical practice have been captured, the completeness and scope of data illustrate how a shift in practice has occurred. This study also highlights how new practice can precede guideline changes.

## 5 CONCLUSION

MRI‐P has affected how clinicians perform cT‐staging. MR visualisation clearly influences reporting of tumour extension. Implementation of prebiopsy MRI‐P has improved agreement between clinical and pathological staging, especially for GG ≥3. This study suggests that MRI‐P affects treatment decision in certain patient subgroups.

## AUTHOR CONTRIBUTIONS

Erik Skaaheim Haug and Christian Beisland are responsible for the study conception and design. Tor Åge Myklebust, Erik Skaaheim Haug and Bjørn Hofmann collected the data. Erik Skaaheim Haug, Tor Åge Myklebust and Christian Beisland analysed and interpreted the results. Erik Skaaheim Haug, Patrick Juliebø‐Jones and Christian Beisland prepared the draft manuscript. All authors reviewed the results and approved the final version of the manuscript.

## DISCLOSURE OF INTEREST

In regard to the present study, the authors have nothing to disclose.

## Supporting information


**Figure S1.** Flowchart showing inclusion and exclusion for the study.

**Figure S2.** Number of MRI‐Ps in Norway 2013–‐2020. These were retrieved from the Norwegian Health Economics Administration (HELFO) for the period 2013‐2020 using coding in the Norwegian Classification of Radiological Procedures (NCRP). In most cases, MRIs performed by private providers were funded by the public health care system.
