# Supplementary figures and images for: Impact of prebiopsy MRI on prostate cancer staging: Results from the Norwegian Prostate Cancer Registry
Source: BJUI Compass. 2023 Jan 10;4(3):331–8. doi: 10.1002/bco2.214 (PMC10071082; doi:10.1002/bco2.214)

**Supplementary Figure 1.**

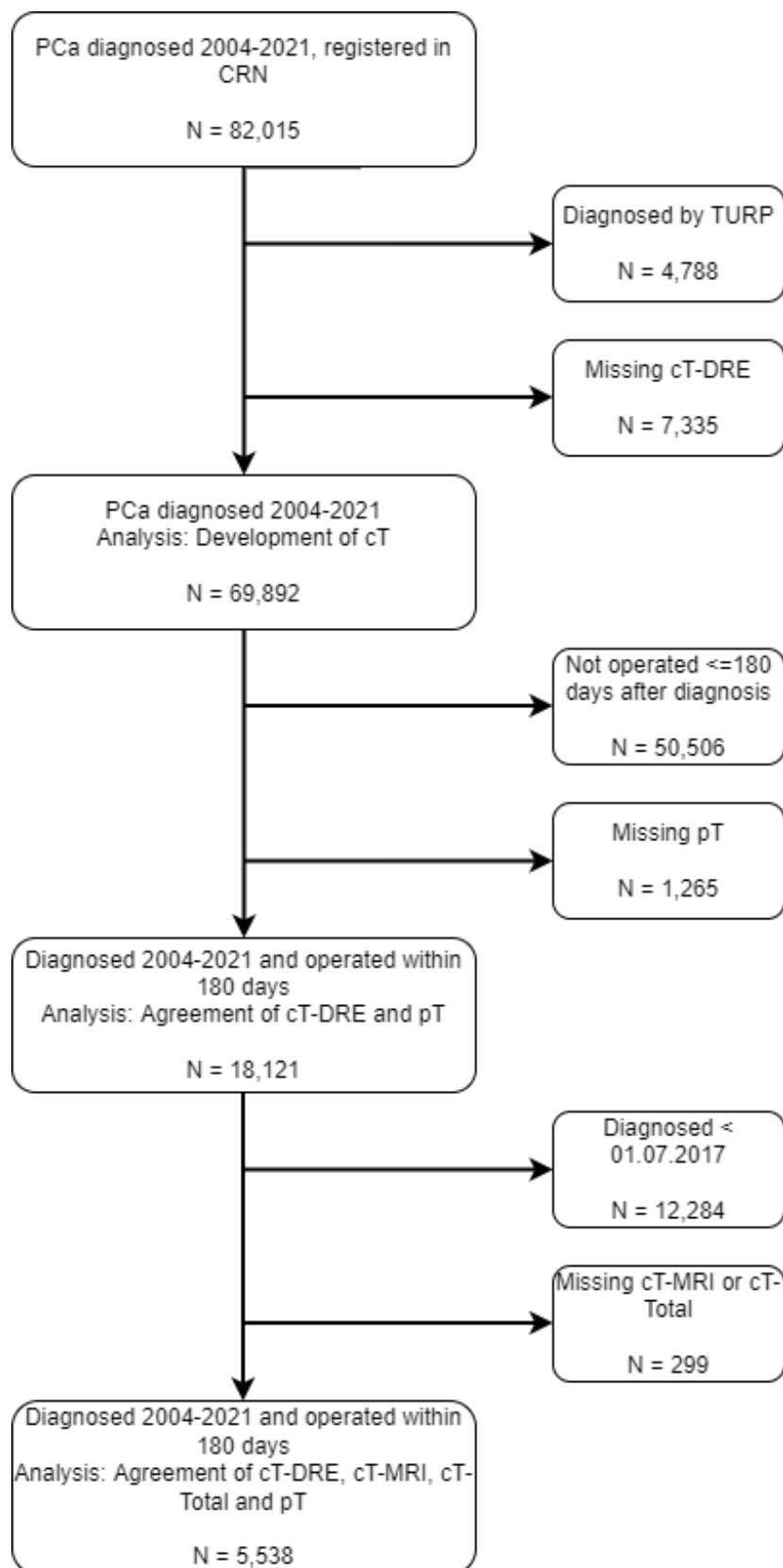

Supplement: Supplementary file 1 — Figure S1. Flowchart showing inclusion and exclusion for the study. [file BCO2-4-331-s002.pdf]

**Supplementary Figure 2.**

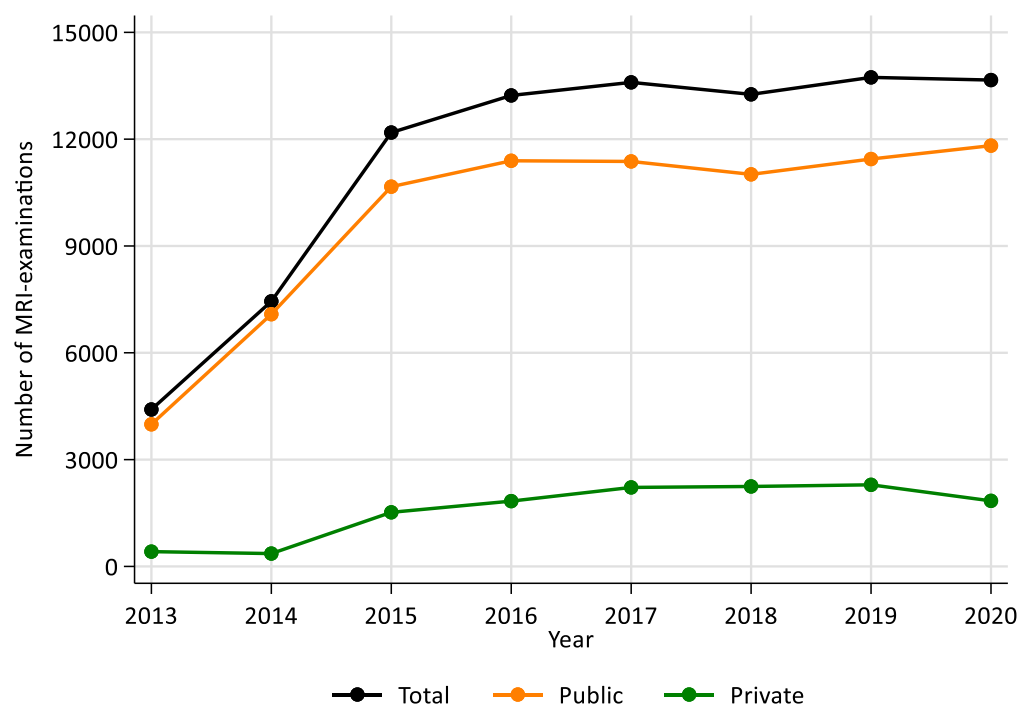

Supplement: Supplementary file 2 — Figure S2. Number of MRI‐Ps in Norway 2013–‐2020. These were retrieved from the Norwegian Health Economics Administration (HELFO) for the period 2013‐2020 using coding in the Norwegian Classification of Radiological Procedures (NCRP). In most cases, MRIs performed by private providers were funded by the public health care system. [file BCO2-4-331-s001.pdf]
